# Rapid Transmission of a Hyper-Virulent Meningococcal Clone Due to High Effective Contact Numbers and Super Spreaders

**DOI:** 10.3389/fgene.2020.579411

**Published:** 2020-12-07

**Authors:** Jonathan C. Holmes, Luke R. Green, Neil J. Oldfield, David P.J. Turner, Christopher D. Bayliss

**Affiliations:** ^1^Department of Genetics and Genome Biology, University of Leicester, Leicester, United Kingdom; ^2^School of Life Sciences, University of Nottingham, Nottingham, United Kingdom

**Keywords:** *Neisseria* meningitidis, meningococcus, meningitis, asymptomatic carriage, transmission, mathematical model, superspreaders

## Abstract

Rapid transmission, a critical contributory factor in outbreaks of invasive meningococcal disease, requires naïve populations of sufficient size and intermingling. We examined genomic variability and transmission dynamics in a student population subject to an 11-fold increase in carriage of a hypervirulent *Neisseria meningitidis* serogroup W ST-11 clone. Phylogenetic clusters, mutation and recombination rates were derived by bioinformatic analyses of whole-genome sequencing data. Transmission dynamics were determined by combining observed carriage rates, cluster sizes and distributions with simple SIS models. Between 9 and 15 genetically-distinct clusters were detected and associated with seven residential halls. Clusters had low mutation accumulation rates and infrequent recombination events. Modeling indicated that effective contacts decreased from 10 to 2 per day between the start and mid-point of the university term. Transmission rates fluctuated between 1 and 4% while the R(t) for carriage decreased from an initial rate of 47 to 1. Decreases in transmission values correlated with a rise in vaccine-induced immunity. Observed carriage dynamics could be mimicked by populations containing 20% of super spreaders with 2.3-fold higher effective contact rates. We conclude that spread of this hypervirulent ST-11 meningococcal clone depends on the levels of effective contacts and immunity rather than genomic variability. Additionally, we propose that super-spreaders enhance meningococcal transmission and that a 70% MenACWY immunization level is sufficient to retard, but not fully prevent, meningococcal spread in close-contact populations.

## Introduction

*Neisseria meningitidis* is present in the nasopharynx of 10–35% of young adults in an asymptomatic ‘carriage’ state (Coureuil et al., 2012). Invasive meningococcal disease (IMD) occurs when meningococci traverse the epithelial and endothelial barriers leading to septicaemia and cerebrospinal meningitis. IMD can be endemic, with rates of ∼1 cases/100,000 individuals, or epidemic, as occurs in sub-Saharan Africa (Caugant and Maiden, 2009). Starting in 2009, the United Kingdom was subject to consistent yearly increase in IMD due to hyper-virulent MenW sequence type (ST) 11 strains (Ladhani et al., 2015). A sub-variant appeared in 2013 and rapidly became the dominant cause of MenW IMD. In 2015, combatting of these infections in the United Kingdom was initiated with provision of MenACWY conjugate vaccines to adolescents and first year university students (Campbell et al., 2015).

An epidemiological study at the University of Nottingham (UoN) in 2015–2016 examined the impact of MenACWY immunization among university students (Oldfield et al., 2017). First-year students (*n* = 1,410) were sampled in September during registration and in five catered halls of residence in November and March. Due to an on-campus catch-up program, MenACWY immunization levels were raised from 31% for in-coming UoN students to 71% (Turner et al., 2017). Meningococcal carriage was detected by performing pharyngeal swabs followed by immediate inoculation of swabs onto GC selective plates and, after 24–48 h growth, selection of oxidase-positive colonies for further analysis (Oldfield et al., 2017 and Oldfield *et al.*, 2018). Colonies were confirmed as *N. meningitidis* by PCR amplification of meningococcal genes and then subject to whole-genome sequencing (WGS) by Illumina HiSeq (125-bp paired end reads and an average coverage of 125-fold). This survey led to identification of 54 carriers of ST-11 MenW clones. Despite the immunization program, MenW carriage increased from 0.7% in September to 6.8% in November and 8% in March [7]. This rapid expansion of a single bacterial lineage, and availability of WGS data, offered an opportunity to examine both the rates of meningococcal genome evolution and the fundamental transmission parameters associated with rapid clonal expansion of meningococcal strains.

## Materials and Methods

### Phylogenetic Analysis

All WGS data is available within Neisseria PubMLST database [available at https://pubmlst.org/neisseria/] (Jolley and Maiden, 2010). Phylogenetic relationships were determined using Genome Comparator, a Bacterial Isolated Genome Sequence database tool, and the *N. meningitidis* cgMLSTv1.0 core genome scheme (Jolley and Maiden, 2010). Genome comparisons and NeighbourNet networks of Nexus distance matrices, weighted by numbers of allele differences, were generated in SplitsTree V4.14.8. Accessory and multi-copy genes were excluded from the phylogenetic analyses.

### Analysis of Mutations and Recombination Fragments

All locus sequences (3,034 loci; 20–5,000 bp) available through Neisseria PubMLST were retrieved, aligned and assessed for frequencies of synonymous and non-synonymous single nucleotide allelic variants using a custom Python (version 3.7.7) pipeline (the pipeline and variable gene sequences for each cluster are available at https://figshare.com/s/d130851f7f9764466161). Alignments with gaps and incomplete or highly divergent alleles were excluded (22–100/cluster). Variant nucleotides were identified by comparison to consensus sequences and tagged to specific codons for detecting synonymous/non-synonymous mutations. Recombination fragments and biases due to two allelic variants in one codon were avoided by limiting analyses to genes with single variant nucleotides.

Recombination in whole genome alignments was investigated with RDP4 (v.4.97; Martin et al., 2015). Contigs were re-ordered with ABACAS (Algorithm Based Automatic Contiguation of Assembled Sequences v1.3.1), using default match, gap and substitution values (Assefa et al., 2009), relative to the closed genome sequence of *N. meningitidis* strain M25419 (CP016678.1; BIGSdb id 47007). Genome sequences for each phylogenetic group were aligned in Mauve (version 20150226 build 10; Darling et al., 2004) and trimmed with trimAl (v.1.4; Capella-Gutiérrez et al., 2009). Recombinant loci were detected by interrogating sequence alignments of 3,061 loci (as defined in the PubMLST Neisseria database) for single variant nucleotides. Loci with truncated alleles or identity thresholds below 90% were excluded. Recombinant loci were defined for each cluster by the presence of one or more alleles whose sequences differed from the consensus sequence by two or more nucleotides.

### SIS Model of Transmission

Transmission parameters were derived by applying an SIS model to published carriage estimates. A Markov chain was developed in R (R version 3.6.1) and applied to determine how individuals transitioned between three mutually exclusive nodal states; susceptible, infected and vaccinated. Our assumptions were of a constant population, homogeneous mixing of individuals, stochastic starting conditions, and linear movement through states. States were defined as two dependent variables - I(t), number of infected individuals at time t, and V(t), number of effectively vaccinated individuals derived assuming 79% efficiency of prevention of carriage acquisition for vaccinated individuals (Cohn et al., 2017), – and a single independent variable S(t), the number of susceptible individuals at time t. The total population (N) was defined by N = I(t) + S(t) + V(t), where a susceptible individual converts to an infected individual through nasopharyngeal strain acquisition, remains infected for a period of time (i.e., is a carrier) followed by strain clearance and return to the susceptible state (i.e., protection against further carriage was ignored; Allen and Burgin, 2000; Huppert and Katriel, 2013; Miller, 2017).

One key parameter is “force of infection” (*ß*), which is derived from the number of contacts per person per unit time between a susceptible and infected person across which transmission occurs. The dynamics of spread were quantified through two ordinary differential equations which describe the real time force of infection across the population per time period:

S(dt)/t(dt) = – ßIS/N

I(dt)/t(dt) = ßIS/N – γI

where *γ* = 1/D and D is length of carriage, calculated to be 5.5 months from a literature survey (Hethcote, 2000; Caugant et al., 2007). Using epidemiologically based estimates of carriage rate per month, numbers of susceptible individuals per unit time were determined from S = N – (V + I). N was the total student numbers in the study population and V was obtained by multiplying observed immunization rates (i.e., 31% for in-coming students and 70% following immunization of 39% of in-coming students) by an estimate of the efficacy of the MenACWY vaccine for prevention of the acquisition of carriage (i.e., 79%; Cohn et al., 2017). As vaccinees were assumed to require ≈1 month to develop full immunity, V was set at 24.5% of students in September rising to 55% in November.

As our population consisted of both susceptible and non-susceptible hosts, we calculated effective reproductive numbers, R(t), for meningococcal carriage with R(t) being defined as the average number of infections caused by an infectious individual occurring over the total infective period. We note that R is extrapolated from transmission data at a specific time point and hence can change over time as the transmission data changes. R(t) was calculated by extrapolation from the cases/month to the average carriage period of 5.5 months using the equation R(t) = beta/0.182^∗^(S/N).

The force of infection contains two sub-terms – the effective contact number per person and the probability of transmission. The effective contact number is a combination of contacts where transmission occurs plus contacts where transmission fails but was possible. Effective contacts were derived from *λ* = ß/T where T is the probability of transmission from an infected to a susceptible person. T was calculated by rearranging the equation to T = ß/*λ*. These values were determined from models assuming random mixing in one hall of residence over 6 months and employing previously estimated rates of physical contact (Mossong et al., 2008).

## Results

### Association of MenW Clonal Clusters With Residential Halls

**FIGURE 1:**
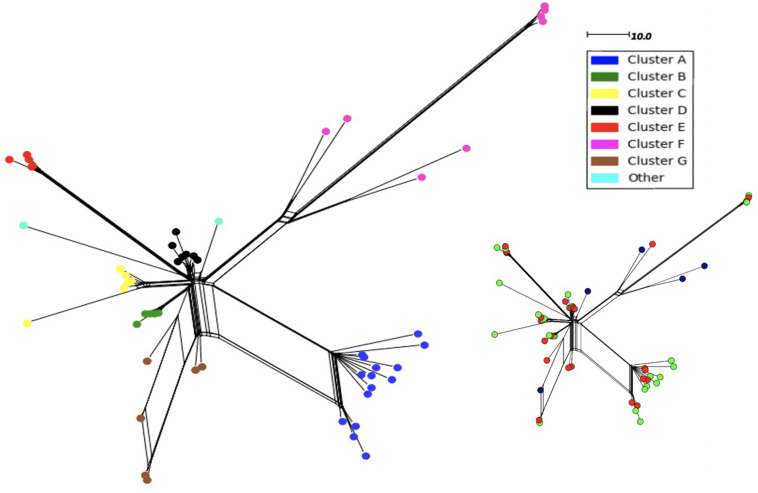
Neighbor-net phylogenetic tree of 54 MenW ST-11 meningococcal genomes obtained over one academic year from students within the University of Nottingham. Isolates were obtained at three time points: September (*n* = 5), November (*n* = 24), and March (*n* = 25). Phylogenetic trees were derived using the PubMLST *N. meningitidis* cgMLST v1.0 scheme. Nodes are color coded based on a classification of an individual isolate into 1 of 7 clusters or no cluster. The scale denotes the number of loci with a variant allele sequence. The same tree is shown as an inset with the isolates color-coded for time of isolation:- blue, September; red, November; green, March.

In order to determine whether the 11-fold expansion of the *N. meningitidis* MenW ST-11 clone among UoN students was due to localized clusters and rapid genome evolution, we analyzed WGS data for the 54 MenW isolates obtained from this cross-sectional carriage study (Oldfield et al., 2018). Analysis of 3,034 core genomic loci identified 603 gene alleles with 1–2 variant nucleotides (see SNP_Analysis folder available at https://figshare.com/s/d130851f7f9764466161). Genetic variability in these carriage isolates is similar to that seen in United Kingdom disease isolates from the same clone and time period (see Oldfield et al., 2018). Phylogenetic trees derived with these sequences indicated that 96% of isolates (52/54) were separable into seven clusters (A–G) of between 5 (B and E) and 15 (A) isolates (Figure 1). Each cluster contained at least 2 isolates from the November and March time points while September isolates were only found in clusters F and G or as singletons. Cluster F consisted of 8 original sub-clone isolates of the MenW-cc11 lineage while all other clusters contained 2013 sub-clone isolates. Clusters A, F, and G had high within-cluster divergence with ∼300 variant alleles between some cluster F isolates.

**FIGURE 2:**
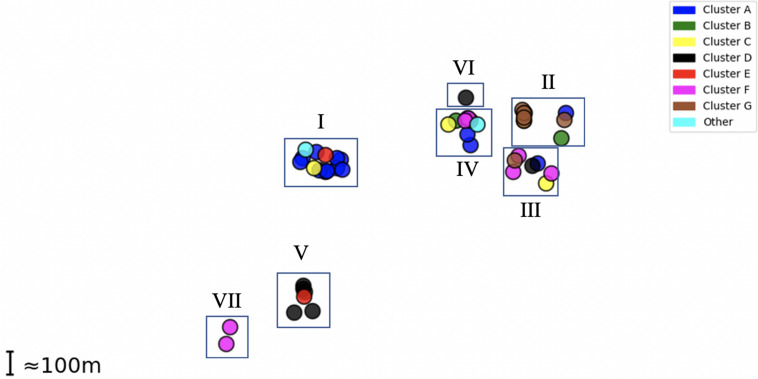
Distribution of MenW isolate clusters throughout the UoN campus. Each point represents an isolate while clusters are color-coded as in Figure 1. Halls I-VII (*n* = 7) are shown as rectangles in their relative positions.

Epidemiological sampling in November and March encompassed students residing in seven halls of residence. Each cluster contained isolates from two or more halls of residence (Figure 2 and Table 1). A Chi-Square test of independence for uniformity generated a high P value (3.9e^–06^) enabling rejection of the null hypothesis that location has no influence on cluster formation. Trends for association of clusters with specific residential halls are exemplified by 67% of cluster A and 57% of cluster D isolates occurring within one hall (Figure 2). These findings indicate that there was rapid, highly localized spread of genetically distinct clones.

**TABLE 1:** Numbers of synonymous and non-synonymous allelic variants, recombinant fragments and recombinant loci in meningococcal clusters.

Cluster (Isolates)	Variant loci^1^	Non-synon (Ka)	Synon (Ks)	Ka:Ks Ratio	Recombination fragments^2^	Recombinant loci^3^
A (15)	90	61	29	2.10	10	21
B (5)	6	4	2	2.00	0	9
C (6)	18	9	9	1.00	0	18
D (7)	15	11	4	2.75	0	6
E (5)	8	7	1	7	0	0
F (8)	270	98	172	0.57	17	69
G (6)	48	16	32	0.5	0	25
Total	455	206	249	0.8	27	148

*^1^Variant loci were identified by analysis of 3,034 loci defined in the Neisseria PubMLST genome repository and are defined as having one variant nucleotide relative to the consensus sequence of all isolates within a cluster. ^2^Recombination fragments were predicted using RDP4. ^3^Recombinant loci, loci where one or more alleles contained > 1 variant nucleotides relative to the consensus of all alleles within a cluster.*

### Expanding Clones Evolve by Mutation and Recombination

The extent and directionality of evolution associated with meningococcal transmission was evaluated by identifying core genome loci containing single polymorphic sites for each cluster, thereby avoiding variation arising by horizontal gene transfer, and the cumulative numbers of synonymous (K_*s*_) and non-synonymous (K_*a*_) variants. The total K_*a*_/K_*s*_ ratio, representing variation between clusters, shows a slight bias towards non-synonymous mutation whereas individual clusters have a wide range of K_*a*_/K_*s*_ ratios (Table 1). Clusters D and C have markedly divergent ratios indicative of very differing selection pressures.

Mutation rates were estimated from observed within-cluster allelic variation and assuming cluster initiation from a single clone. High numbers of allelic variants in clusters F and G are not compatible with a single origin and were excluded from subsequent analyses. Numbers of single allele variants for the 38 isolates of clusters A to E were averaged across the population. Assuming expansion from a single isolate and a standard power expansion model, rates of variant allele acquisition were linear with only a minor decrease over time (data not shown).

Putative horizontal gene transfer events for each cluster were predicted by detection of recombination fragments with RDP4 and by counting recombinant loci with > 1 variant nucleotide (Table 1). Numbers of recombinant loci exceeded recombination fragment numbers by 5-fold. The former is probably an overestimate as multiple loci within recombination fragments are split apart while RDP4 underestimates recombination by imposing higher minimum variant nucleotide thresholds for each sequence window. Nevertheless, multiple recombination events were detected in clusters A and F, possibly due to these clusters having multiple founder organisms. Four clusters contained multiple putative recombinant loci, but no recombination fragments, while no evidence of recombination was detected in cluster E. Ratios of mutant and recombinant loci (ranging between 0.25 and 1.5) were similar for clusters A, D, F, and G indicating that both processes contributed to cluster evolution while cluster E was only evolving through mutation.

### Evidence for Initiation of Clonal Clusters by Single Entrant Carriers and Derivation of Transmission Parameters

**FIGURE 3:**
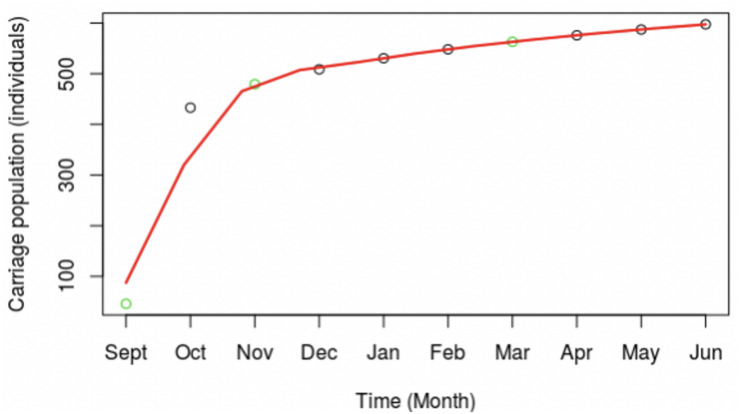
Expansion in carriage of the MenW:ST-11 lineage at the University of Nottingham for the 2015–2016 academic year. The overall carriage dynamics were extrapolated from three observed time points using a power expansion model. Actual numbers of carriers were determined from observed carriage rates for three time points (indicated by green dots). A power expansion was utilized to calculate the carriage rates and total numbers of carrier (black circles) for other time points. A line of best fit shown in red was fitted to the observed and estimated carriage numbers. Carriage estimates were based on a fixed population size of 7,049, the total number of first year students at UoN.

Exploration of the meningococcal transmission parameters was initiated by extrapolating from our published MenW carriage data (see Introduction) to the whole first year student population of 7,049 individuals. An estimate was derived for the total MenW carrier numbers for this specific academic year group using a natural log expansion model (green circles, Figure 3). The model shows an initial, rapid 10-fold increase over 2 months followed by a slow, modest expansion phase (Figure 3). In a power expansion model, the initial expansion occurs mainly within month one (data not shown).

A key rationale for the UoN study was to test how MenACWY vaccination influenced spread of the hypervirulent MenW clone. Our modelling assumed that 31% of vaccinated entrants were fully immune to disease while those immunized in September, a further 39%, developed full immunity to disease by October. As MenAWCY vaccines have reduced efficacies, for preventing meningococcal carriage (estimated as 79% and contrasting with 100% protection against disease; Cohn et al., 2017), we estimated that the percentages for carriage-immune vaccinees (V) was 23.7% in September and 55.3% from October onwards (see Table 2) with other individuals being either carriers (I) or susceptible to carriage (S).

**TABLE 2:** Outputs of an SIS model of carriage dynamics for a first-year student cohort.

Time	V^1^	S^1^	I^1^	ß^1^	R(t)^1^	*λ*^1^	T^1^
Sept	1726	5277	46	11.48	47.23	10.3	0.04
Oct	3898	2718	433	0.75	1.58	1.29	0.02
Nov	3898	2672	479	0.65	1.34	1.93	0.01
Dec	3898	2642	509	0.60	1.23	2.24	0.01
Jan	3898	2621	530	0.58	1.19	2.22	0.01
Feb	3898	2603	548	0.57	1.15	2.62	0.01
March	3898	2588	563	0.56	1.13	2.13	0.01
April	3898	2575	576	0.55	1.10	2.47	0.01
May	3898	2564	587	0.55	1.09	2.43	0.01
June	3898	2554	597	0.55	1.09	2.41	0.01

*^1^V, vaccinated individuals; S, susceptible individuals; I, carriers; ß, force of infection; R(t), effective reproduction number; *λ*, effective contacts/day (30.5 days per month); and T, transmission rate/effective contact.*

Transmission rates were estimated from a model of random mixing among a homogenous population. Cluster A had a high association with one hall (Table 3) and was selected as being representative of clonal expansion within a residential hall of an average size of 265 as the numbers of isolates obtained in the November and March time points, 4 and 6, respectively, were in line with the predictions from the overall carriage rates (i.e., 4.1 and 4.8 obtained using the observed carriage rates of 6.7% and 8%, respectively, and a 23% sampling size for the hall). Carriage rates were estimated for this single hall with the model curve obtained in Figure 3 and assuming 2 initial carriers (i.e., Figure 1 indicates that cluster A comprises two clusters initiated from different founder organisms). Effective contacts per unit time were derived from the contact patterns of 15–19 year olds described by Mossong et al. (2008). Taking physical contacts as being those where transmission can occur across a 55% physical contact rate (an average of home and work physical contact rates) with half of the daily contact rates occurring within the hall, we estimated that a hall of residence had average daily and monthly contact rates per person of 4.8 and 147.

**TABLE 3:** Distribution of MenW clusters between halls of residence.

Hall	Cluster							Total
	A	B	C	D	E	F	G	
I	10	0	1	0	1	0	0	12
II	1	2	2	0	0	0	5	10
III	1	0	2	2	0	3	1	9
IV	3	1	1	0	2	1	0	8
V	0	2	0	4	2	0	0	8
VI	0	0	0	1	0	1	0	2
VII	0	0	0	0	0	3	0	3
Total	15	5	6	7	5	8	6	52

Modelling with the observed carriage rates and our initial estimate of effective contacts generates transmission rates per effective contact of 4% in September decreasing to 1% in February with a slight rise in later months (Table 2). Major trends in transmission rates are, as expected, reflected in the other interlinked parameters with initially high rates for *λ*, ß and R(t) followed by a steady decline and a final minor upward surge in May. Interestingly numbers of effective contacts for individuals begin at ∼10 per day before decreasing to ∼1 per week by October. Slight increases in transmission rates in later months compensate for the cumulative effects of loss of carriage in a large population.

In order to control for sample size effects, we also ran models using the numbers of carriers estimated from the upper and lower 95% confidence intervals as reported by Oldfield et al. [26]. The R(t) values were 34 and 207 in the first time point for the upper and lower carriage estimates, respectively, but were similar to our previous values for other time points (see carriage estimates file available in the SIS_Model folder at https://figshare.com/s/d130851f7f9764466161).

We also considered that hall A may not be representative of other halls or the wider university population. We therefore examined models of across university spread with a fixed number of effective contacts (73.5, 147, and 294) or fixed transmission rates (0.04 and 0.002) with values predicated on data from our initial model (Table 2). Fixing the effective contacts provided upper and lower bounds for the transmission rate as 0.002 and 0.156. Contrastingly, the low transmission rate produced very high levels of effective contacts but a rate of 0.04 indicated that realistic effective contact numbers of 287/month (9.4/day) in September and dropping to ∼14/month (0.45/day) in later time points.

### Impact of Super-Spreaders on Carriage Rates

**FIGURE 4:**
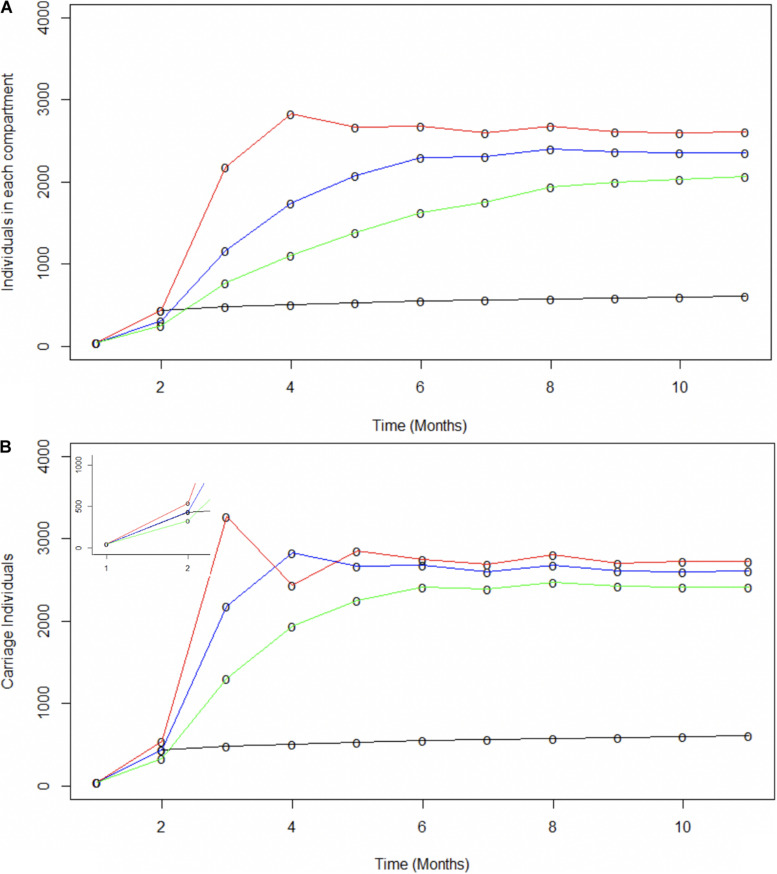
Modeling the potential contributions of super-spreaders to carriage dynamics. The contributions of super-spreaders (SS) to carriage dynamics were tested by running SIR models with either varying effective contact ratios for a fixed proportion of SS or varying proportions of SS with a fixed ratio of effective contacts. **(A)** indicates the impact of differences in the ratio of effective contact rates for SS vs. nSS. This model was run with 20% of the carriers being SS. The SS:nSS effective contact rate ratios were:- red line, 1000:147.5; blue line, 500:147.5; green line, 250:147.5. **(B)** displays the impact of variations in the proportion of SS. These models utilized a constant effective contact rate of 1000:147.5 for SS to nSS, respectively. The relative proportions of nSS to SS were: - red line 70:30; blue line, 80:20; and green line, 90:10. The inset displays the data for months 1 to 2 on a broader scale. In both panels the observed carriage is indicated by the black line.

Spread of specific disease-causing meningococcal clones may be facilitated by hyper-transmissible strains, with an enhanced ability to transmit between hosts, or super-spreaders, whose behavioral patterns have many more contacts resulting in increased spread for the same bacterial transmission rate (Galvani and May, 2005; Lloyd-Smith et al., 2005). Super-spreaders were investigated by splitting populations into two sub-populations termed super-spreaders (SS) and non-super-spreaders (nSS). Models investigated differences in transmissibility levels for SS and in proportions of SS. Transmissibility levels were modelled as differing effective contact rates (Figure 4A). As these rates change over time, the ratio of effective contacts per month for nSS versus SS was fixed. For this model, an 80:20 SS:nSS proportion was adopted based on the pareto principle (Stein, 2011). Model outputs were compared to observed carriage rates and similarities to all carriage expansion phases (Figure 4A). The only phase accurately captured by these models is the initial one with the best fit for this early phase being a 1000:147.5 ratio wherein the 20% SS population accounts for 87% of all infections. Lack of fit derives mainly from significantly higher or lower carriage plateaus and lower initial carriage due to fixed contact rates. Modelling of a range of proportions of SS for a fixed 1000:147.5 nSS:SS effective contact ratio showed that a 30% or 10% SS proportion exceeded or undershot the initial expansion phase, respectively, but again significantly over-estimated the carriage plateau (Figure 4B). While these models indicate that fixed ratios are a poor measure of overall transmission, we can conclude that a realistic number of super spreaders (i.e., 20%) could have underpinned the rapid, initial clonal expansion of MenW ST-11 clones within the UoN population.

## Discussion

Highly transmissible strains are putative drivers of United Kingdom and global increases in MenW IMD. Stemming from high numbers of effective contacts and diverse strain types, the close contact environments of universities facilitate rapid strain spread and heighten risks of meningitis outbreaks. Here we studied the rapid amplification in carriage of hypervirulent MenW clones in a partially-immunized student population.

Utilizing data from a previous cross-sectional carriage study (Oldfield et al., 2017), we estimated that there were 46 carriers of MenW cc11 isolates among the 7,049 UoN entrants (0.7% MenW carriage rate; 769 September swabs). Sampling in November and May (353 and 288 swabs, respectively) from seven halls (representing 1,855 entrants; 265/hall) detected an 11-fold increase in MenW carriage. A phylogenetic analysis indicated the presence of seven divergent clonal groups and two singletons. Adoption of a higher stringency for definition of a clone (<20 divergent core genes from the common ancestor) splits groups F and G into 6/7 clonal groups and group A into two separate clones. Thus, we have detected between 9 and 15 separate clones or 1.28-2.1 clones/hall, which is close to the estimate of 1.75 clones/hall derived assuming an even distribution of MenW entrants among halls. This indicates that clonal expansion from single entrant carriers is a major mode of meningococcal transmission.

Importantly carriage clusters mapped closely to residential locations within UoN, and not to other features such as sampling time point, indicating a high frequency of expansion of meningococcal clones within catered university halls of residence. Whilst biased sampling, due to peer pressure resulting in multiple samples from friendship groups, may influence cluster size, our data indicate that each cluster arises due to spread from one entrant to 15–44 individuals (an extrapolation from observed cluster sizes of 5 to 15 with a 34% sample size and assuming some spread between residential halls). Whether a similar pattern of spread might be observed in non-catered university halls or within other student networks remains to be determined.

Clonal expansion may exert strong selective pressures due to differing selection pressures between hosts and bottlenecks associated with transmission. Mutation rates varied between 4.8 and 0.24 mutations/genome/month. These mutation accumulation rates are similar to those observed for longitudinal meningococcal carriage (Lamelas et al., 2014; Pandey et al., 2018; Green et al., 2020), indicating that transmission does not impose a strong selection for elevated fixation of mutants or for higher mutability. Putative recombination fragments were found in almost all clusters at varying frequencies, with greater detection in the diverse phylogenetic clusters (i.e., A, F, and G) potentially reflecting divergence among founder clones. For clusters with a single putative founder (i.e., B, C, D, and E), mutation and horizontal gene transfer generated similar numbers of variant genes (47 and 33, respectively) with an average of 20 variable genes per cluster. This latter rate is only 2.8-fold higher than occurs during persistent carriage in an individual (Green et al., 2020) and implies that rapid spread of meningococcal clones in a naïve population does not require significant levels of genetic variation.

Two important measures of disease potential are R_0_, basic reproduction, and R, effective reproduction, numbers, that relate to spread in fully or partially immune populations, respectively (Guerra et al., 2017; van den Driessche, 2017). MenC infection was estimated to have a low R_0_ value of between 1.2 and 1.36 while R(t) was estimated to fluctuate between 0.87 and 1 (Trotter et al., 2005), however these measurements were based on countrywide carriage rates and disease case data and a low MenC carriage rate of 1% (138/14,057 in 1999; Maiden et al., 2008). Hence these studies may have underestimated transmission dynamics arising from asymptomatic carriage within a close contact environment of a more frequently carried strain. By focusing on spread of a single clonal lineage within a university setting, we estimate that the R(t) for asymptomatic carriage fluctuates between 47 at the start of term and 1 in later periods. The initial high value is comparable to viral R(t) numbers and is indicative of a highly transmissible organism. Notably, this occurred despite a 30% immunization rate. Intriguingly 85% of the MenW isolates were from the 2013 MenW lineage and this lineage was the main driver of the clonal expansion whereas the original MenW ST-11 lineage isolates (cluster F) were present in similar numbers and percentages at all time points (i.e., 3, 2, and 2 isolates and 0.4%, 0.6%, and 0.6% carriage rates for the September, March, and December time points, respectively). Thus, these data suggest that high rates of transmission between carriers have driven 2013-associated IMD outbreaks, such as the one associated with an international scout convention (Lucidarme et al., 2016), and increasing incident rates of this sub-clone both within the United Kingdom and other countries. We note that these sub-lineages are differentiated by a small number of recombination events and differing lengths of phase-variable repetitive tracts that may be determinants of transmissibility (Lucidarme et al., 2016 and Green et al., 2019).

A novel observation was of the relationship between rapid initial meningococcal spread and high, but realistic, numbers of effective contacts. These high values reflect risk factors associated with increased social mixing and behaviors likely/known to occur during the initial months of a university term and previously associated with meningococcal transmission. Notably, actual transmission values are relatively low with transmission occurring in 1–4% of effective contacts. The major drop in effective contacts that occurs between September and October could be due to reduction in social mixing as individuals settle into defined social groups or the rising immunity prevents transmission. A caveat to this speculation is that the reproduction numbers, effective contacts and transmission rates are derived from curves fitted to observed data and are interlinked due to the nature of SIR models. Thus, changes in one value will have a compensatory impact on the other values. Nevertheless, these values provide a basis for designing controlled transmission studies and/or more detailed epidemiological studies of a wider range of meningococcal clonal complexes and transmission settings. We also observed that a highly mixing subpopulation (super spreaders), with a 7-fold higher proportion of effective contacts, could replicate observed clonal expansion. Because all other individuals have low numbers of effective contacts, increases in carriage are almost entirely due to the super-spreaders. One caveat is that super spreader behavior may be particular to university settings and may not be constant. Two other considerations are that super spreaders may have intrinsically higher carriage densities, and hence higher transmission rates (as opposed to higher numbers of effective contacts), or alternatively that some meningococcal clones have higher transmission rates from all carriers.

**FIGURE 5:**
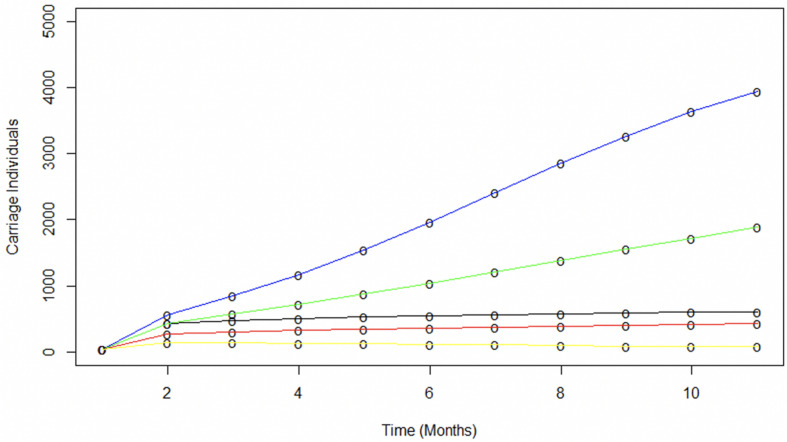
Modeling the contribution of various vaccination scenarios on meningococcal spread. The impact of various vaccination scenarios were modelled using average transmission parameters derived previously in Table 2. The blue line indicates the spread of MenW in a completely naive population. No uptake of vaccine from the start of term is modelled by the green line. A 70% vaccination uptake (red) and a 100% vaccination proportion (yellow) shows the impact of vaccination prior to the start of term. Finally the observed carriage is indicated by the black line.

Another critical observation was that the 40% increase in MenACWY conjugate vaccine uptake at the start of the UoN term produced a significant decrease in susceptible contacts and limited MenW spread. MenC and MenY conjugate meningococcal vaccines are known to produce herd protection (Maiden et al., 2008) but the effect of MenW conjugate vaccines on carriage was unclear due to observations of MenW carriage in vaccinees (Oldfield et al., 2018). Figure 5 shows that the 70% vaccine coverage achieved in UoN at the start of term produced a significant reduction in spread and the carriage plateau as compared to a population with no vaccine coverage or with only the 30% coverage of the entrant population (i.e., the situations prior to introduction of the vaccine and occurring in universities without catch-up campaigns, respectively). This figure also shows that a 70% or 100% entrant coverage rate would have produced further reductions in carriage levels, indicating the potential impact of the adolescent immunization campaign. In summary, our study shows that moderate and high levels of vaccine coverage can halt rapid spread of MenW strains between carriers and suggests that MenACWY conjugate vaccines can produce herd protection, a key goal of the current UK MenACWY immunization strategy.

While our findings may be specific for this lineage and setting, predictions of high R(t) values for carrier-to-carrier spread of meningococcal clones is a novel insight into the importance of asymptomatic carriage as a critical determinant of disease frequency for bacterial pathogens where a carrier state is a key life cycle stage. Our study also raises the scenario of university students being a major source of national and international spread of meningococcal clones as students are likely to carry clones back into their home communities.

## Data Availability Statement

Meningococcal genomes sequences and read data are available within NeisseriaPubMLST (https://pubmlst.org/bigsdb?db = pubmlst_neisseria_isolates). Other raw data supporting the conclusions of this manuscript will be made available by the authors, without undue reservation, to any qualified researcher.

## Ethics Statement

The studies involving human participants were reviewed and approved by Nottingham University Medical School Ethics Committee. The patients/participants provided their written informed consent to participate in this study.

## Author Contributions

CB and JH conceived the design of the mathematical analysis. LG and JH performed genome sequence analyses. JH performed the mathematical modelling. CB, NO, and DT designed the carriage study. CB and JH drafted the manuscript. All authors contributed to the article and approved the submitted version.

## Conflict of Interest

CB reports grants from GlaxoSmithKline, Sanofi Pasteur MSD, and Roche, outside the submitted work. The remaining authors declare that the research was conducted in the absence of any commercial or financial relationships that could be construed as a potential conflict of interest.
